# Ultrasonic Weld Quality Inspection Involving Strength Prediction and Defect Detection in Data-Constrained Training Environments

**DOI:** 10.3390/s24206553

**Published:** 2024-10-11

**Authors:** Reenu Mohandas, Patrick Mongan, Martin Hayes

**Affiliations:** 1 Department of Electronic and Computer Engineering, University of Limerick, V94 T9PX Limerick, Ireland; martin.j.hayes@ul.ie; 2 Confirm Smart Manufacturing Research Centre, V94 T9PX Limerick, Ireland; patrick.mongan@ul.ie; 3 School of Engineering, University of Limerick, V94 T9PX Limerick, Ireland

**Keywords:** weld strength prediction, autoencoder, weld defect detection, convolutional autoencoder (CAE), ultrasonic welding (USW)

## Abstract

Welding is an extensively used technique in manufacturing, and as for every other process, there is the potential for defects in the weld joint that could be catastrophic to the manufactured products. Different welding processes use different parameter settings, which greatly impact the quality of the final welded products. The focus of research in weld defect detection is to develop a non-destructive testing method for weld quality assessment based on observing the weld with an RGB camera. Deep learning techniques have been widely used in the domain of weld defect detection in recent times, but the majority of them use, for example, X-ray images. An RGB image-based solution is attractive, as RGB cameras are comparatively inexpensive compared to X-ray image solutions. However, the number of publicly available RGB image datasets for weld defect detection is comparatively lower than that of X-ray image datasets. This work achieves a complete weld quality assessment involving lap shear strength prediction and visual weld defect detection from an extremely limited dataset. First, a multimodal dataset is generated by the fusion of image data features extracted using a convolutional autoencoder (CAE) designed in this experiment and input parameter settings data. The fusion of the dataset reduced lap shear strength (LSS) prediction errors by 34% compared to prediction errors using only input parameter settings data. This is a promising result, considering the extremely small dataset size. This work also achieves visual weld defect detection on the same limited dataset with the help of an ultrasonic weld defect dataset generated using offline and online data augmentation. The weld defect detection achieves an accuracy of 74%, again a promising result that meets standard requirements. The combination of lap shear strength prediction and visual defect detection leads to a complete inspection to avoid premature failure of the ultrasonic weld joints. The weld defect detection was compared against the publicly available image dataset for surface defect detection.

## 1. Introduction

Welding is a universally used technology for joining structural components in aircraft, automobiles, machines, construction, pipelines, etc. The technology and equipment used for welding differ based on the type of welding joints that adhere to a welding standard. Defects in welding are a natural cause for concern, and non-destructive testing (NDT) methods for automatic weld defect detection technologies have been an emerging area of research [1]. The most widely used non-destructive inspection (NDI) techniques include visual testing by a qualified operator [2,3], radiographic testing involving X-ray or gamma rays to generate weld joint images that reveal internal defects [4], ultrasonic testing involving high-frequency sound waves to detect flaws in weld joints [5], magnetic particle testing, which observes the behaviour of applied magnetic particles to detect surface and near-surface defects [6], and liquid penetrant testing, which involves the usage of penetrating fluid that seeps into cracks or pores and further examining the area under UV light after removal of the fluid [7]. Magnetic methods like eddy current testing are one of the promising techniques in NDT applications for steel since the system configuration is simple, fast, and non-contact [8]. Another electromagnetic inspection technique, alternating current field measurement, has the added advantage of determining defect size (length and depth) in a wide range of structural materials [9]. In the weld defect testing procedure, using X-ray or gamma rays produces a weld radiographic image by first generating the weld structure and then exposing the photographic film. The defect in the weld joint is identified by a change in intensity in the radiographic image. These films are inspected by a certified operator, which is a time-consuming and tedious process in the mass production environment [1]. The authors in [10] observe visual inspection as a rudimentary method that requires an experienced human. However, recent research has developed algorithms to automate this process and perform it more accurately and efficiently than humans.

The automation of weld defect detection is a significant step in manufacturing. A real-time weld defect detection process allows dynamic corrective measures to overcome the defect or halt the welding process to avoid further wastage [10]. These are essential steps to avoid damage to the welded structure, thus possibly avoiding critical safety hazards. In recent years, automated defect detection systems have advanced in manufacturing. Image processing techniques with feature extraction and classification have been widely used in weld defect detection from radiographic images [11].

Further advancement of AI-based systems has led to the use of deep learning techniques for weld defect detection [2]. This work analyses the encoding of image data using a CNN-based autoencoder [12] to engineer a predictive analysis of weld quality in a manufacturing setting. When the data available is limited, and the cost of building a comprehensive dataset is prohibitive, data generation techniques can be used. For example, data augmentation techniques and multi-modal data fusion can be used to overcome data scarcity [13] and train deep learning models. Computer vision-based techniques with pre-processing steps for feature engineering and extraction were used for weld defect detection from X-ray images in [10]. These techniques were further employed in multiple research avenues involving red-green-blue (RGB) images, but few are publicly available to the research community. The defects produced in a weld strongly depend on the fused materials and the type of welding technique used [14,15]. Hence, the defects are also different for each problem. Weld defects can be categorized into two types: external and internal. Weld cracks, undercuts, spatter, porosity, overlap, and craters are external welding defects. Slag inclusion, incomplete fusion, necklace cracking, and incompletely filled grooves are internal welding defects [16].

**Figure 1:**
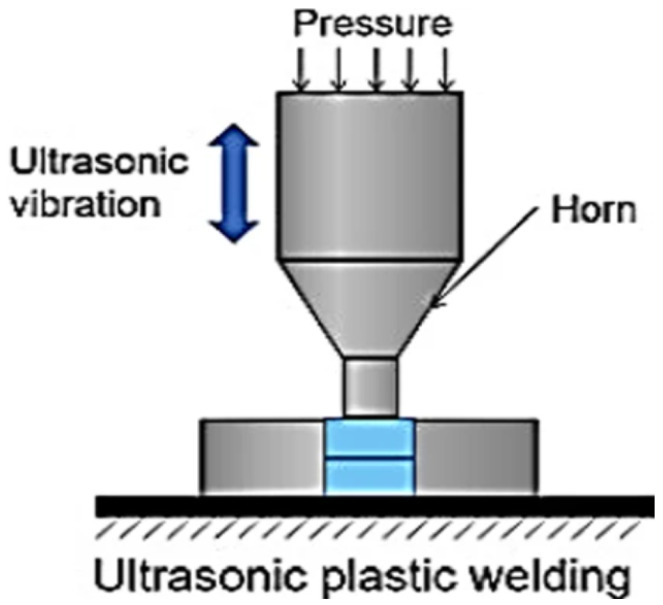
Diagrammatic representations of the USW process, adapted from [18].

Ultrasonic welding (USW) is an important method for joining plastics and is a significant method for welding polymer composites [17]. USW is a specific focus for this paper. Figure 1 is a diagrammatic representation of the USW process. Thermoplastics are light and cost-effective, which makes them highly popular in, for example, the automotive sector to reduce the weight and cost of vehicles [18]. The plastic welding process involves heating the plastic components until they are flexible and then joining them. Plastics start to fuse at the moment of melting. They will be entirely fused only when they are cooled. Hot gas welding, laser beam welding [19], spin welding [20], vibration welding [21], hot plate welding [22], and high-frequency [23] and solvent welding [24] are all processes used for the process of plastic welding. In USW, high-frequency, low-amplitude mechanical vibrations are used to generate the friction required to generate the heat [18].

Due to its adaptability to various polymer composites, short process times, low energy requirements, and ease of automation, USW is preferred for mass-production applications [14]. However, the integrity and strength performance of the joint are significantly influenced by the weld process input parameters [14,18]. Frequently, multiple inspection methods are used to determine the weld joint quality. For example, joints can have good lap shear strength (LSS) but a lot of matrix degradation due to excessive heat during welding, leading to premature failure. To address this issue, we propose two methods: weld strength prediction and visual defect detection.

**Figure 2:**
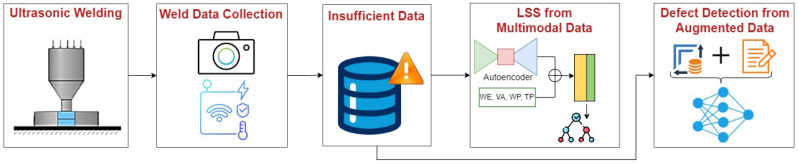
USW quality inspection from constrained dataset.

Figure 2 gives an overview of the stages of development of the two-step weld joint inspection experiment presented in this paper. This work focuses on the performance improvement brought forward by the fusion of image data with input settings data acquired under the same manufacturing setting to predict the strength of weld joints, considering LSS as the quality attribute. To that end, we developed and used a multimodal dataset containing input parameter settings and encoded image data of the weld joints. The sample size obtained was 28. A convolutional autoencoder (CAE) was designed and trained to extract image feature data.

Along with weld quality prediction, high-accuracy weld defect detection is also proposed in this work. For this task, a dataset of weld joint images showing the welding defects (the USW weld defect dataset) is set up using offline image augmentation techniques. This dataset is annotated into three different classes based on the severity of visual defects. This annotated USW defect dataset is then used to train computer vision deep learning algorithms to detect weld joint defects. This work proposes two quality checks that can be performed on the same weld joint for a complete inspection to mitigate premature failure.

1. First, LSS evaluation is performed to ensure the strength of the joint is within acceptable limits.
2. Then, an automated assessment of visual defects in the joint is executed to determine if any defects will lead to premature failure.

Using computer vision in the loop for real-time product quality control poses many challenges. As with all deep learning methodologies, algorithms are only as good as the data they are trained on [25]. The problem of imbalanced or limited datasets hinders the development and deployment of AI-based solutions on the factory floor. Conversely, optimised manufacturing processes greatly reduce the occurrence of faulty samples. Developing models on small datasets is a common issue for engineering applications, and this study is no different. The objective is to produce parts that exhibit a target LSS value for the USW joint to minimise destructive testing, thereby increasing production efficiency and lowering production costs. As we will show later in this paper, the weld defect detection achieves an accuracy of 74%, which is >70%, the reference industrial floor rate of accuracy set by the manufacturer in cellular manufacturing processes (a production strategy aiming to increase efficiency on the production floor continually) [26]. In cellular manufacturing implementation, a 50% or more improvement on any performance metric is categorised as a successful implementation [26].

The primary contributions of this work are as follows:

- This work proposes a complete two-stage inspection procedure to determine weld joint quality, comprising strength prediction from a multimodal dataset and visual inspection from an augmented image dataset.
- Development of a multimodal dataset containing encoded image data and input parameter settings data to reduce prediction errors in weld quality prediction.
- Demonstrated improvement in USW weld strength prediction using a multimodal dataset. This work illustrates a 34% reduction in prediction errors when using multimodal data.
- Presentation of a novel CAE design that can efficiently extract encoded image data when the dataset available is extremely small.
- A benchmark comparison of weld defect detection methods on the developed USW defect dataset against publicly available surface defect detection dataset methods is provided.

This paper is organised as follows. We provide background information and a literature review in Section 2; the proposed deep learning-based method is discussed in Section 3; the experimentation and results are described in Section 4; and conclusions are drawn in Section 5.

## 2. Background and Literature Review

Surface defect detection is a significant step in quality analysis in industrial manufacturing [27]. It is one of the industry’s most scrutinised specifications, making it critical that manufacturers and consumers adhere to the same prescribed standards to ensure high quality in manufactured products. Deep neural networks have been used in various real-time manufacturing applications and have drawn significant interest from across the machine learning community [28].

The quality and quantity of data produced by Internet of Things (IoT)-enabled devices in smart manufacturing environments continue to increase rapidly. With the widespread use of sensors comes a large inflow of data (e.g., time series or discrete digital data), often stored in large databases. To utilize big manufacturing data, advanced data analytics is increasingly needed to process the high volume, high velocity, and wide variety of data collected [29]. Deep convolutional neural networks (CNN) are commonly used to extract relevant features and patterns from the massive volume of labeled and unlabeled data [30].

In the smart manufacturing paradigm, manufacturing machines are fully monitored by sensors and dynamically controlled by intelligent, real-time systems to improve product quality, productivity, and sustainability [29,30]. Mass production assembly lines were deployed with the idea that every individual did their part in the process to manufacture good quality products [30]. Despite the emergence of dynamic smart manufacturing systems, manual quality control is a challenging and principal financial burden. The rate of defects in some industries have been estimated to equate to half the production rate, and even 90% in more complex manufacturing lines, as estimated by the European Commission [29].

The current trend of the manufacturing revolution focuses on using intelligent systems to provide high-quality products while increasing productivity and reducing production costs [30]. Deep learning is extensively used in two main areas of manufacturing: inspection and defect detection. Defect detection is an important step in the industrial manufacturing process, and CNNs have been widely used in defect detection due to their powerful feature extraction capability [31]. One specific problem is the lack of balanced training data. There should be a sufficient representation of true and false positives for a representative number of different use case settings [32]. Even with the improved computational capabilities of IoT-enabled sensors, curating a sufficiently balanced training dataset can present a significant challenge. Importantly for the work proposed in this paper, techniques such as transfer learning, fine-tuning, and data augmentation have ensured higher model accuracy [33].

### 2.1. Weld Defect Detection

As mentioned previously, welding is an important technology in many industries. Weld quality should be ensured according to the welding standards; hence, weld defect detection has become an emerging research topic over the years [34]. Papageorgiou et al. [35] observed zero defect manufacturing (ZDM) as one of the important strategies in the Industry 4.0 framework. Traditional machine learning methods, such as Bayesian networks, logistic regression, K-nearest neighbour, support vector machines, etc., have all been used in industrial operations and defect detection. AI-based ZDM technologies have advanced in the past decade. Computer vision-based automated technologies have been used in manufacturing. These belong primarily to four different categories [25]:

- Dimensional quality, where the dimensions of the object under inspection are assessed to determine if they are within specific tolerances.
- Surface quality, where the surface is inspected for cracks, wear, scratches, etc.
- Structural quality, where the manufactured component is analyzed for the presence of unnecessary parts, or lack of required components.
- Operational quality, where the quality of the object is inspected to determine if it is fit for the required purpose.

The weld defect detection methods proposed in this paper use RGB images rather than the more common X-ray images. In addition, we propose the use of the four input parameters of the welding process. This work shows that combining visual information with input setting information can significantly reduce errors in machine learning-based weld quality prediction compared to using input settings alone.

**Figure 3:**
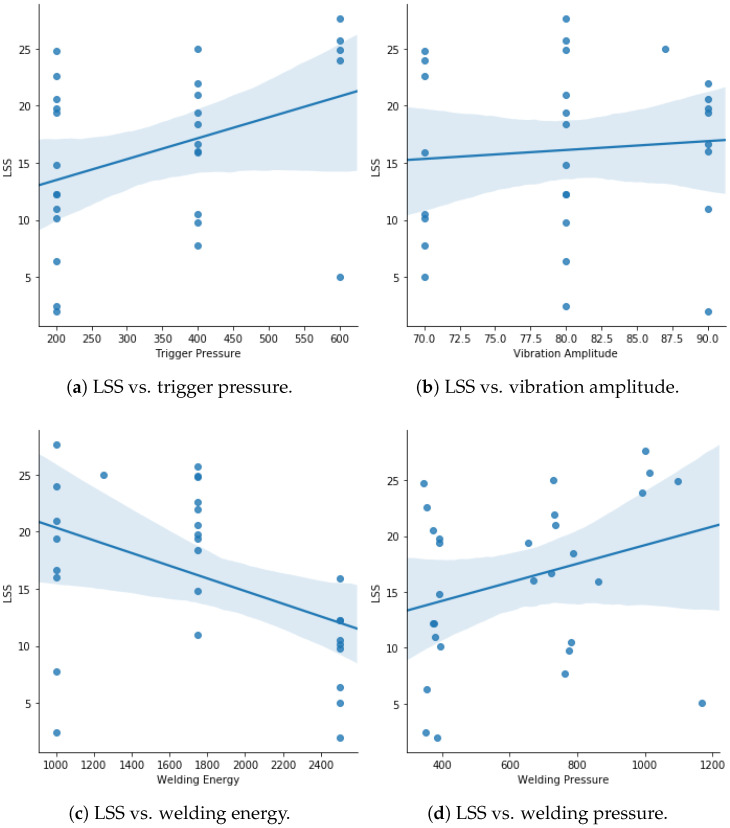
Comparison of LSS against (**a**) trigger pressure; (**b**) vibration amplitude; (**c**) welding energy; and (**d**) welding pressure.

**Figure 4:**
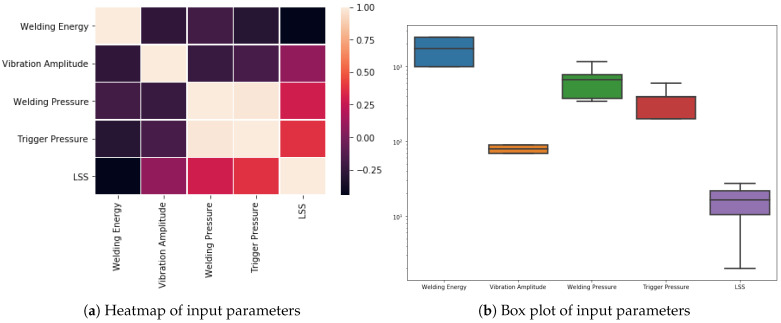
Plot (**a**) shows the heatmap of all the four input parameter settings, and (**b**) shows the box plot indicating the range of values for the input parameter settings.

Figure 3 shows the comparison of weld joint strength measured as LSS against input parameters: trigger pressure (Figure 3a), vibration amplitude (Figure 3b), welding energy (Figure 3c), and welding pressure (Figure 3d). Mongan et al. [14] observed that vibration amplitude influences joint LSS the most. However, the graphs in this paper indicate that an increase in trigger and welding pressure has an incremental effect on joint LSS. Welding energy, however, has a reverse effect on the joint LSS. From the above observations, it is clear that all four parameters significantly affect LSS, but not in a linear manner. This informs the motivation to use machine learning regression techniques to predict the LSS of the weld joint; machine learning algorithms are powerful feature extractors from raw data [14,15,36]. Figure 4a shows the heat map of input parameters, which indicates that welding energy and vibration amplitude have the greatest influence on LSS. Figure 4b shows the range of the input parameters and LSS.

Sassi et al. [37] used the deep learning network DenseNet [38] to develop automatic weld defect detection using a dataset of 378 labelled images. Using a small dataset, Le et al. [39] used an ensemble model for surface defect detection. Their originally available defect images were 50 defective images of decorative sheets and 56 defective images of welding joints. They used random imitation and Wasserstein GANs [40] for data augmentation and a multi-model ensemble network containing Inception V3 [41], MobileNet [42], and other CNNs for defect detection. Gao et al. [43] investigated real-time defect detection for industrial applications and tested their methodology on the defect datasets NEU-DET [44] and DeepPCB [45]. They used ResNet50 as the backbone, a fully convolutional neural network for expanding the receptive field for feature information collection, and Gaussian weighted pooling as the region of interest approach. Based on all of the complex combinations of experiments, they also observed the lack of precisely annotated datasets for surface defect detection as the motivation for using feature information extraction and digital signal processing techniques to improve the accuracy of deep learning techniques.

Yang et al. [46] observed that weld defect recognition is mainly divided into feature-based and deep learning-based methods. Feature-based methods use image features from X-ray or RGB images, though they have very weak texture characteristics and weak contrast for effective image feature extraction. Previously, sensors such as vision sensors [47], infrared sensors [48], ultrasonic sensors [5], and X-ray sensors [49] have been used for welding defect detection. Duan et al. [49] proposed an automatic weld defect detection from X-ray images using an adaptive cascade boosting (AdaBoost) algorithm for classification. Roy et al. [50] used time-frequency domain signal processing methods such as discrete wavelet transform to detect defects in friction stir welding and verified the internal defects in weld samples using computed tomography scan images. Weld bead inspection using a structured light-based vision inspection system was proposed in [51]. The study presented the dimensions of the weld beads, image processing, and extraction algorithms for laser profiles for defect detection during multi-layer welding processes.

Chen et al. [52] proposed a weld defect detection method using the selective reconstruction of background and weld regions in test images such that the defective regions would be suppressed by sparsity reconstruction. Computing the difference between the reconstructed and test images highlighted the defective regions. Dong et al. [53] performed weld segmentation and defect detection on X-ray images using traditional image processing techniques to automate the weld defect detection process.

Among the deep learning-based defect detection techniques, intelligent process control for laser welding has been studied in [54]. The authors used a deep autoencoder to extract low-dimensional features from the high-dimensional image data. The extracted features were then fed into a temporal-difference learning algorithm to acquire data and temporal predictions, which were then combined with deep learning to map sensor data directly to the quality of a weld seam. Yang et al. [55] proposed a weld joint detection and identification method based on a deep convolution neural network (DCNN). A deep learning-based surface defect inspection system was proposed by Yang et al. [56]. The authors applied background segmentation and template-matching techniques to determine the region of interest, which was then uniformly cropped into several patches. These patches were fed into a pretrained CNN-based model for classification. The CNN-based model used in this experiment is SqueezeNet [57]. Li et al. [58] proposed an end-to-end surface defect recognition system based on symmetric surround saliency maps for surface defects and a deep CNN for classifying seven categories of steel strip defects. Fu et al. [59] developed a surface defect classification method using the SqueezeNet baseline (SDC-SN-baseline) on the NEU-DET (North Eastern University) detection benchmark dataset. They fine-tuned low-level features in the pretrained model to better characterise texture-related defects on steel surfaces. The authors also incorporated multiple receptive fields to improve the distinctiveness of high-level features. The weld defect detection method proposed by Tripicchio et al. [25] focused on using computer vision techniques and deep learning to cope with unforeseen changes in production quality. They used pre-filtering and a weighted confusion matrix to avoid retraining and obtain good performance estimations.

The challenge addressed in this work is the limited dataset. The original number of images available was 28, which is extremely low in terms of building a dataset. Input parameter setting values corresponding to each weld joint were also available, enabling data fusion. Data augmentation was applied as a data-space solution to address the problem of limited data [60]. We used these methods to overcome the limited data problem. The focus of this work is to identify a model that is readily deployable in a real-time manufacturing setting and that is optimal in relation to the mitigation of these factors. In particular, any misclassification that might occur concerning minor/cosmetic versus major defects has significant ramifications for parts being incorrectly assigned to an unnecessary process step. This analysis aims to identify the model that ensures that the correct downstream action, which may include further human involvement in the detection process, is always taken.

### 2.2. Autoencoders

**Figure 5:**
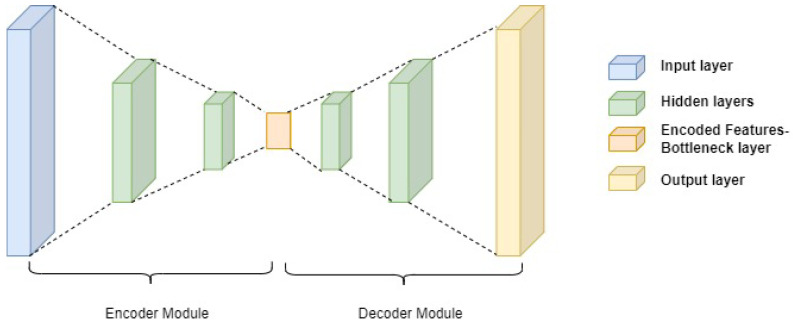
An autoencoder block schematic diagram showing the encoder module, the decoder module, and the bottleneck layer with reduced dimensionality.

Autoencoders are generative models trained to reconstruct input data [61]. The architecture of an autoencoder network consists of an encoder module and a decoder module (Figure 5); that is, they are unsupervised learning algorithms with symmetrical structures. An autoencoder network is trained to map an input to its output, but is designed to be unable to generate a perfect copy of the ‘real’ input [62]. The encoder part compresses input data into lower dimensional data with distinct informative content. The decoder module then uses these extracted features to reconstruct the input data [63]. The premise is that, upon completion of training, the encoder module could be used as a powerful feature extractor. Autoencoders have been widely used in detecting anomalies through training on normal data; abnormal inputs can be identified when the reconstruction error produced is higher than that of the normal inputs [64].

Autoencoders were initially suggested as a novel way of modelling sensor data, so that the use representative synthetic data could overcome the challenges posed by a lack of actual data [62]. A simple autoencoder consists of a feed-forward neural network consisting of one input layer, a hidden code layer, and then the output layer [62]. They are restricted to learning to approximate the input so that the network can prioritize those features that should be copied to represent the input in a compressed state. Thus, they have often been used for dimensionality reduction or feature learning, which can be attributed using principal component analysis (PCA) [32]. More recently, they have been applied to sensor data modelling [65]. Autoencoders can capture characteristics of the internal structure of input data to regenerate them at the output. The compressed information at the bottleneck layer is rebuilt by the decoder using the loss function to update weight parameters so as to reduce reconstruction errors [66]. Deep autoencoders can learn complex hierarchical features using their non-linear representational layers, and are becoming increasingly common for anomaly detection in various fields because of the inclusion of a synthesized reconstruction step. Confidence in the process is established through the measurement of reconstruction error. This metric is used in [64] to detect and label sensor input abnormalities and thus increase detection rates.

The autoencoder used in this study is a convolutional autoencoder (CAE). Convolutional layers have long proven to be better feature extractors from images [63,66]. The encoding is a process by which a deterministic mapping relationship is established from the input to re-extract the data using a specific encoding. The decoding process then converts the encoding back into the input data with minimal reconstruction error. Here, we have designed and trained a CAE for encoded feature extraction. Dimensionality reduction was also achieved by extracting the encoded features. The dimension of the bottleneck layer for effective feature encoding was experimentally determined. The experiments used to determine the dimension of the code layer and number of epochs for training the autoencoder are discussed in Section 3.1.1 and Section 3.1.2. These extracted image features are then used to develop the multimodal dataset. The autoencoder was primarily selected as one of the methods we used to address the problem of limited data. Data augmentation techniques were also used to generate sufficient data to train the autoencoder.

## 3. Methodology

**Figure 6:**
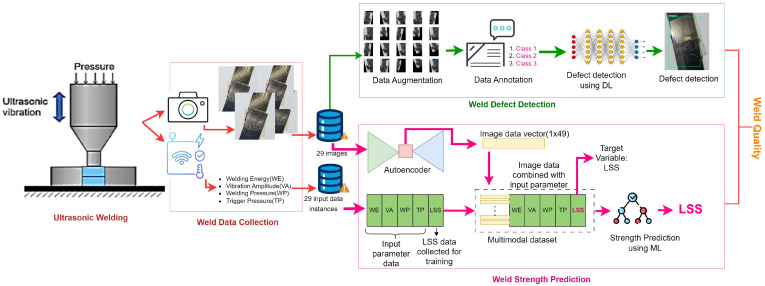
Process flow for a complete weld quality inspection, including both strength prediction and visual defect detection to mitigate premature failure of the weld joint.

This work addresses two important aspects: weld strength assessment by LSS prediction and visual weld defect detection from RGB images using the developed USW defect detection dataset. The combination of these two processes leads to a complete weld quality inspection. The flow of the overall inspection process is given in Figure 6. The weld quality prediction uses an autoencoder to extract image features in a compressed format and fuse the information with sensor input data acquired from the experiment setting. The extracted feature values are fused with the input parameter values (welding energy, vibration amplitude, welding pressure, and trigger pressure) corresponding to each image instance to generate a multimodal dataset. This dataset is then used to train the regression algorithms to predict the values corresponding to the weld joint’s LSS. The novelty of this work lies in mapping input parameters directly to image data, which proves very useful in the prediction of USW joint quality.

The weld defect detection is conducted on the proposed USW defect detection dataset. The instances are divided into three categories, annotated as classes 1, 2, and 3 based on the severity of weld defect. Due to the limited number of data samples, image augmentation was used to increase the number of image data instances. The USW defect dataset was also evaluated against a publicly available surface defect detection dataset (NEU-DET [44]) to analyze the performance of out-of-the-box deep learning computer vision algorithms on such constrained datasets.

The following sections explain in detail two experiments encountered in this body of work: weld strength prediction from the multimodal dataset, and weld defect detection using computer vision on the USW defect detection dataset.

### 3.1. Experiment: Weld Strength Prediction

Here, we describe the methods we used to predict weld strength, explaining the design of CAE in Section 3.1.1 and the process of multimodal data fusion in Section 3.1.3.

#### 3.1.1. Design of Convolutional Autoencoder

**Figure 7:**
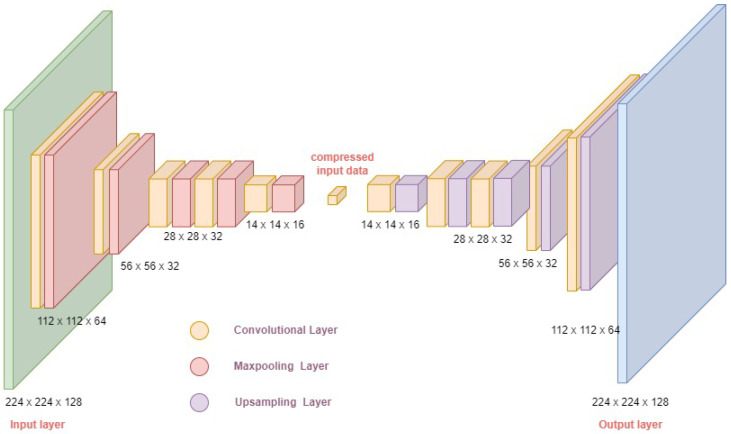
The architecture of the convolutional autoencoder used in this work.

Before prediction, image features were extracted using the CAE network, shown in Figure 7, which used image data in the input and output layers. The input, hidden, and output layers were constructed using CNNs, hence the term convolutional autoencoder. The convolutional layers extracted feature information from the input image, and the activation function was assessed using a rectified linear unit (ReLU) to avoid vanishing gradients. After each convolutional layer in the encoder module, a max pooling layer was added to down-sample the input data into compressed, reduced dimensionality data at the bottleneck layer. In the decoder module, the compressed features were up-sampled using the up-sampling layer to restore the image to its original input size per the algorithm outlined in [67]. The size and number of filters in each layer on the proposed CAE were determined through experimentation. The autoencoder designed and used in this study was a symmetric network with 128 filters in the first input layer, for an image input size of 224 × 224. The final output layer also had 128 filters, with the reconstructed image size as 224 × 224.

In an ideal case, an autoencoder aims to perfectly reconstruct the input data at the output layer, or a near-perfect output with the lowest reconstruction error. A large model capacity is essential for such low-error reconstruction. However, in the use case of autoencoders for feature extraction, it is essential that the autoencoder does not learn to identically reconstruct the original image, which is described as an ‘over-fitting towards identity function’ [68].

In this work, the trained autoencoder was used for extracting feature values, which were later used to synthesize the hybrid dataset with image feature data and input parameter settings corresponding to the conditions that resulted in the weld joint depicted by the image. Hence, from a total of 28 images, 468 training images and 118 test images were generated. There were fewer chances of overfitting at the autoencoder level, as these images were only used in training the autoencoder, and were not further used in the regression stage. The input image was resized to 224 × 224 and then compressed by the encoder network to generate a feature vector of size 7 × 7. With the current vector size of 7 × 7, the number of extracted elements from a single image instance came to 49 numerical values arranged as a row vector. This step was repeated for each of the 28 images. The final data obtained were integrated with the input parameter data columns to complete the multimodal dataset.

#### 3.1.2. Latent Space Representation

The CAE was applied for dimensionality reduction, as the non-linear layers enabled reduced representation after retaining the most relevant information from the input data. In the autoencoder, information passed through the encoder layer until it reached the bottleneck layer (code layer), with the latent space defined by neurons in the bottleneck (code) layer [69]. PCA is the classical method for dimensionality reduction in machine learning experiments. The advantage of autoencoders over PCA is that autoencoders provide a non-linear transformation into the latent space, whereas PCA results in a linear transformation [69]. Autoencoders map the higher dimensional data representation into a lower dimensional latent distribution. The decoder model learns to sample the latent distribution to generate the input data. After completing the training process, the latent space contains information about every instant of the input dataset that is unavailable in the high-dimension representation [70].

In the work [71], the information bottleneck is used to determine compression in the input data.

The information plane of autoencoders suggests that ideal autoencoders with large bottleneck layer sizes do not effectively compress information. Autoencoders with smaller size bottleneck layers cause information compression in the encoder layers [72]. The bottleneck layer is generally the last layer of the encoder. From the theoretical analysis followed in [71], consider that input information is represented by *M*, *K* is the bottleneck layer size, and λ(K) is the maximum amount of information transferred through K. In the case where *K* is small (λ< *M*), information compression occurs to some extent, such that the encoder can hold the allowed information λ at the bottleneck layer. Hence, when the information compression measure is high, and the bottleneck layer size is too small, perfect reconstruction is not possible, and it also leads to information loss, resulting in poor image reconstruction at the output. Hence, an optimal bottleneck layer size of 7 × 7 was used in this experiment.

**Figure 8:**
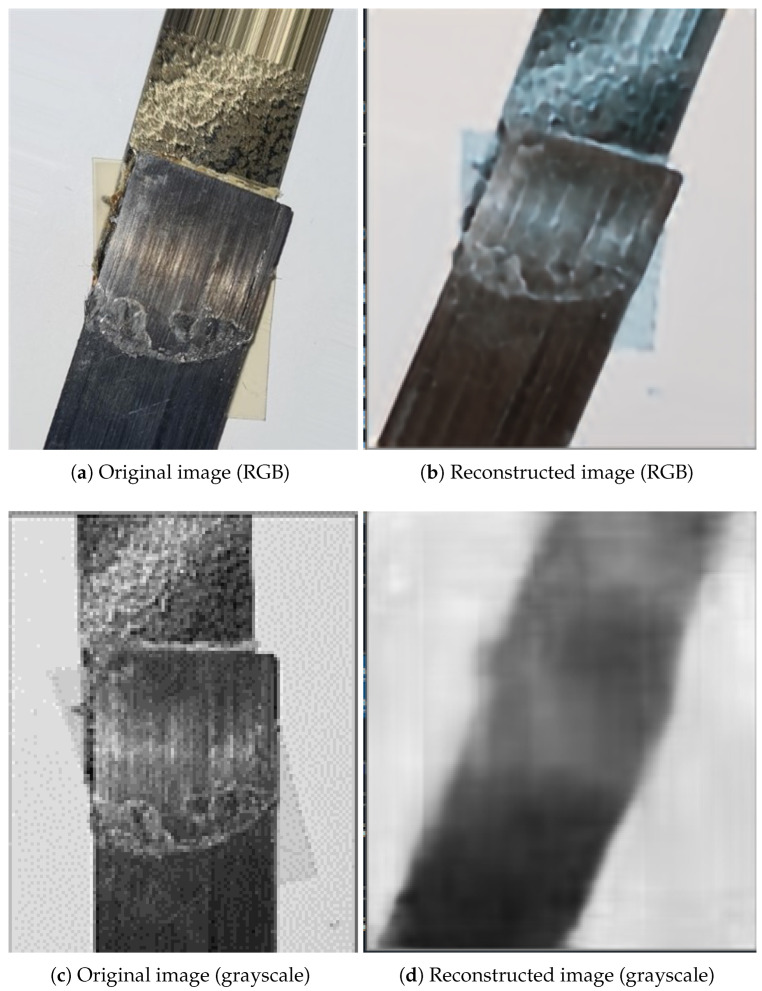
Comparison of an original input image and the reconstructed output image form an autoencoder. Structural similarity of (**a**) vs. reconstructed image (**b**): 0.82, and structural similarity of (**c**) vs. (**d**): 0.67.

The training of the autoencoder was assessed by the quality of the reconstructed image obtained at its output layer. The similarity of the input and reconstructed images at the output was calculated to evaluate the training of the autoencoder. The structural similarity score of the images was calculated using SSIM [73,74], the structural similarity index. SSIM is a widely used image fidelity measure in traditional image processing techniques that evaluates the similarity between the structural elements of the images and their ground truth. Figure 8a,b show the given input as an RGB image from our dataset with higher dimensional data for reconstruction. The reconstruction of the image is very good, with a high structural similarity of 82%. In our case, the bottleneck layer had a higher dimension of 56 × 56, and the network was trained for 100 epochs. When compressed to a 7 × 7 dimension, the RGB image again had an actual vector size of 7 × 7 × 3, which amounted to 147 values, again representative of high dimensional data.

The current study involved reducing the dimension of the input image. Hence, the image was converted into grayscale before feeding into the deep learning network. With reduced dimensionality, the reconstruction was not as good as the higher dimensionality image. This, in turn, accounted for extracting only relevant features for the reconstruction of the input image. Figure 8c,d show a comparison of the original input image vs. the reconstructed image when the bottleneck layer dimension was reduced to 7 × 7, and the input image was grayscale. The reconstructed image was far from perfect, with a lower similarity score of 67%. This is attributed to further compression of image data at the encoding layer. Hence, all the images were converted into grayscale for the autoencoder training.

From Table 1, it is experimentally found that the optimal number of training epochs for the autoencoder for a better final prediction value is 80. At 100 epochs, the mean absolute error MAE) and Root mean squared error (RMSE) at the final prediction tend to increase, which might indicate a case of learning close to the identity function in the autoencoder model. The CAE thus designed for this problem was deep enough to generalize the features and extract only the relevant information to be further used in the machine learning tasks. This is further exemplified by the decrease in prediction error value while using the image data combined with the input parameter data for the USW joint quality assessment.

#### 3.1.3. Multimodal Dataset

**Figure 9:**
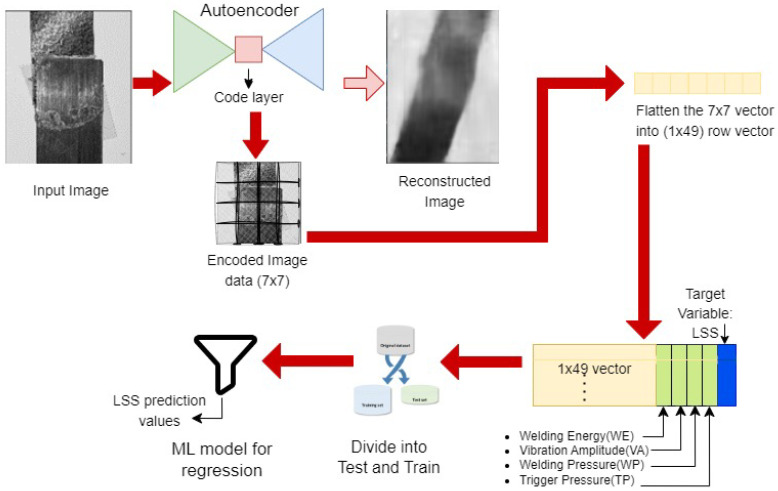
Diagrammatic representation of fusing encoded image data with input parameter data for LSS prediction. A 1 × 49 vector from the autoencoder latent space is appended with the four input variables to create the dataset.

A multimodal dataset is generated from the fusion of multiple modalities such as images, text, audio, and numerical or behavioural data [75]. Multimodal data fusion can be achieved by one of three approaches: early fusion, late fusion (decision-based fusion), or intermediate fusion. This experiment used early fusion, fusing data before the machine learning task. In the proposed method, image data was fused with input parameter settings data obtained for generating the explicit weld joint given in the image (see Figure 9). The image data was in RGB format, and the input parameter settings were sensor data in numerical format. This fused dataset was used to predicts the weld joint LSS.

The multimodal dataset used in this experiment was generated by combining input parameter setting data and image data. This was a multi-stage process. The first stage involved generating the required training data from the limited number of available images using data augmentation. As discussed previously, the next stage was to design an autoencoder with the required input and encoding layer dimensions to convert the images into encoded numerical data with lower dimensions. Once the encoded data was obtained from the trained autoencoder, the final stage was combining the encoded image data with the available input parameter setting data to generate a hybrid dataset which could be used to predict the LSS value.

The dimensions of the original image were 1800 × 4000. This weld joint image was resized to 224 × 224 to match the dimensions of the input layer of the autoencoder used. The number of data instances was 28 images, corresponding to the sensor measurements of welding energy, vibration amplitude, welding pressure, and trigger pressure. The LSS of all these joints were also evaluated, and the values were to be predicted. For the fusion of the dataset, the numerical values from the high dimensional image data were extracted into a 7 × 7 matrix, flattened out to a 49 value vector, and saved in ‘.csv’ format. This data were subjected to normalization using the *MinMaxScaler*, available with *Scikit* learn. This transformed the features by scaling each feature to the range between 0 and 1. The input parameter data obtained were also normalized to ensure compatibility with the extracted image data. This extracted data were integrated with the input parameter data columns to generate a single large dataset of size 28 × 54, including the label column. The data were stored in ’.csv’ format and extracted as a Pandas data frame using Python for further data processing and regression operations. This multimodal dataset was used for the LSS prediction. Regression techniques were applied, including decision tree regressor and random forest regressor. The results are explained in Section 4.3. The data were split into train and test sets in the ratio 70:30 and trained using the regression model selected. The target variable chosen here was the LSS corresponding to the weld joint. The prediction results showed a reduction in error values in the multimodal dataset compared to the sensor dataset. The results obtained are discussed in Section 4.3 and Section 4.4.

### 3.2. Experiment: Weld Defect Detection Using Computer Vision

Besides weld quality analysis, weld defect detection is also an important step in product quality analysis. Defectively welded parts need to be identified and appropriately removed before they are used in automobiles, aircraft, or other important areas of manufacturing. This defect detection differs from a weld quality check because it involves a visual check on the weld joint. This work developed an RGB image dataset with weld joint images to apply weld defect detection using computer vision deep learning algorithms. Despite the increased use of computer vision algorithms to detect and identify weld defects, the number of publicly available RGB image datasets for this purpose is small.

#### 3.2.1. Dataset and Annotation

The total number of image samples obtained was 28, which was extremely limited. The original input image size ranged from 1700 × 2200 after cropping down from the original dimension of 1800 × 4000. Data augmentation was used to overcome the significant challenge of fewer data instances. Augmentation attributes such as rotation, shear, zoom, horizontal flip, and brightness were used to alter the existing images. The total number of final images obtained after augmentation was 586. The Keras Image Data-generator module was used for this offline image data augmentation procedure.

The generated augmented images of weld joints were subjected to defect detection based on the severity of the defect caused during the USW process. The defects were classified into three classes (1, 2, and 3) based on their severity, with class 1 being the least severe/no visible defect and class 3 being the most severe.

For weld defect detection based on images, complex welding processes have a certain effect on the robustness of such algorithms. Hence, image-based techniques are developed for specific use cases and applications in welding [46]. Manual annotation of datasets for deep learning is a laborious and time-consuming task. Collecting image samples from the complex welding environment is also a difficult task. Different welding parameters influence the weld quality, and unbalanced samples in a dataset will affect weld defect detection. The annotation software ‘*labelimg*’ was used to annotate the image sample into classes 1, 2, and 3 for the task of defect detection in the USW defect detection dataset.

#### 3.2.2. Deep Learning Models Used

The deep learning models, SSD-MobileNet [42] YOLOv8x and YOLOv8n [76] were trained for defect detection using the augmented dataset. The primary focus of this experiment was to generate a high-accuracy detection system using a limited dataset. The recommended dataset size for training a detection system is in the range of 10,000 s, with 500–1000 images representing each class. However, this might not be a viable scenario in a manufacturing setting, or in instances where the experiment is a prototype test and limited usage of resources is recommended. Data availability is severely restricted in such cases, and there is little to no access to more data generation under identical conditions and settings. Thus, pretrained models were used for this training, as the limited size of the dataset would not allow for training from scratch. Pretraining improves the generalization capability of deep learning models when the dataset for the task is extremely small [77]. Li et al. [78] have also stated that pretraining is crucial for tasks involving small datasets.

## 4. Experiment and Results

This section discusses the experimental setup for the experiment’s deep learning and regression stages and the software specifications used, including data annotation and augmentation software. The data augmentation techniques used are also explained below.

### 4.1. Experimental Setup

The training of CAE was completed on a GPU GeForce RTX 2080 Ti using TensorFlow and Keras libraries. The regression steps were implemented using *Scikit Learn* libraries. The visual defect detection training step for SSD-Mobilenet was completed on a GPU GeForce RTX 2080 Ti. The TensorFlow version was 1.14, and the detection used Python 3.6 and OpenCV 3.4. The pretrained SSD-Mobilenet model was downloaded from the TensorFlow Model Zoo, and TensorFlow Object Detection API was used for training. The YOLOv8 training was completed using the pre-trained models YOLOv8n and YOLOv8x, downloaded from Ultralytics, Python-3.8.19, torch-2.3.1 on CUDA 12.1, NVIDIA GeForce RTX 3080, 10001MiB.

### 4.2. Data Augmentation

Deep neural networks are invariably used in numerous applications because of their feature extraction power, but are infamous for their data-hungry nature. To prevent overfitting, the number of training samples should be appropriate to the network size and representative of the classes included. This is ensured by using data augmentation techniques [79].

**Figure 10:**
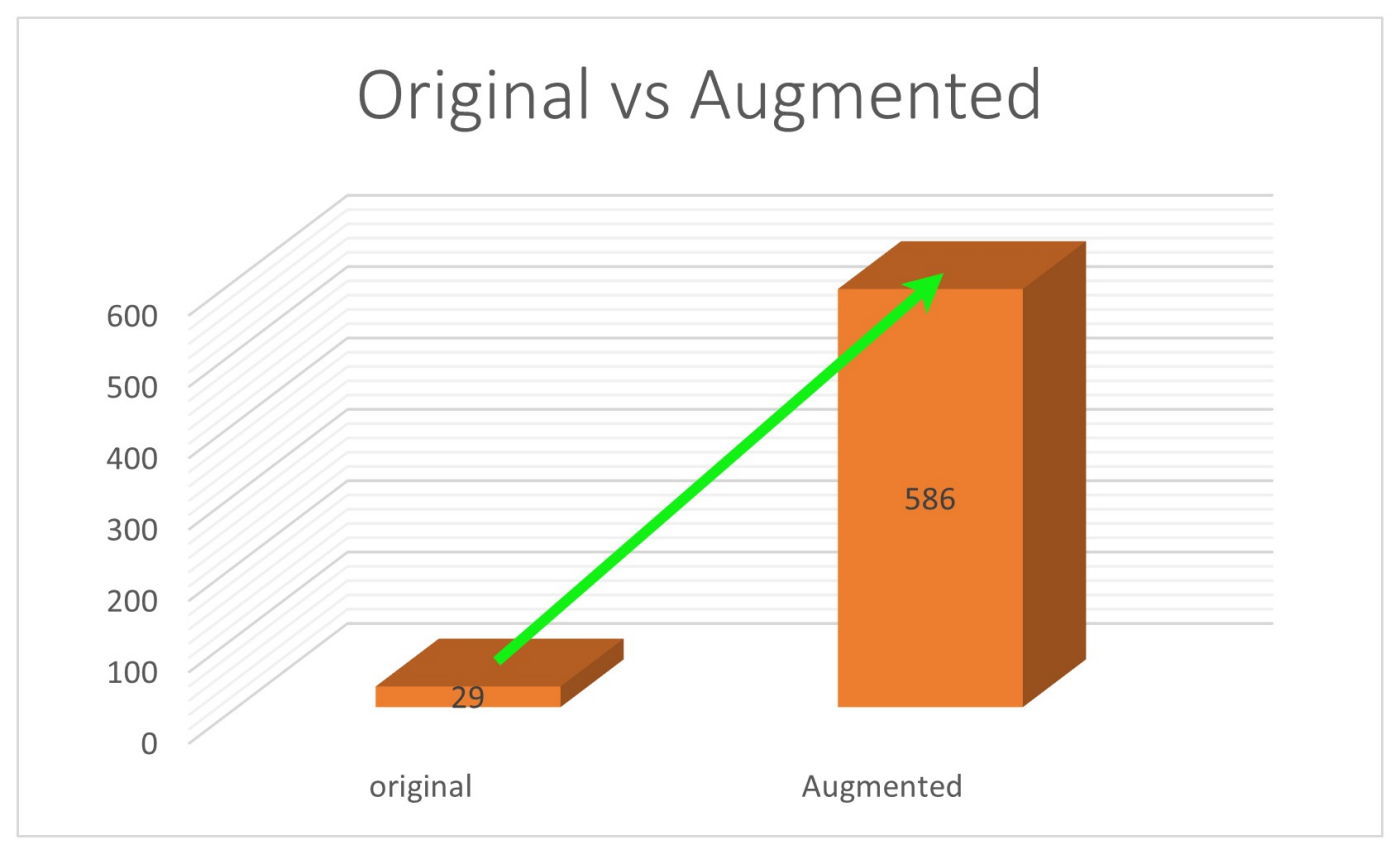
Graphical representation of the increase in the size of dataset: original vs augmented.

The Keras Image Data-generator module was used for data augmentation. The images were subject to rotation, shear, zoom, horizontal flip, and brightness modulations to generate augmented images. This data augmentation method is called offline data augmentation, essential for adjusting the weights for efficient reconstruction. Image augmentations applied to the dataset were rotation, shear, zoom, horizontal flip, and brightness adjustments, before annotation for object detection. After augmentation, the final number of images in the dataset was 586, significantly higher than the original (Figure 10). A further set of data augmentation attributes, ‘noise’ [80] and ‘mosaic’ [81] (both available with YOLO), were also used during the training process.

Training with noise augmentation further improved the accuracy of the USW defect detection dataset. For the larger NEU-DET dataset, further addition of noise augmentation did not significantly impact the training metrics.

The dataset contained 468 train images and 118 test images, divided into three classes. The NEU-DET contains six kinds of typical surface defects of hot-rolled steel strips: rolled-in scale (RS), patches (Pa), crazing (Cr), pitted surface (PS), inclusion (In), and scratches (Sc). The database includes 1800 grayscale images, comprising 300 (split into 240 images for training and 60 for testing) samples per defect category.

### 4.3. Results from Weld Quality Prediction

Weld quality prediction was achieved using the multimodal dataset. This experiment had multiple stages, represented by Figure 9. Firstly, the augmented dataset was used to train the autoencoder. The autoencoder training was assessed based on the reconstruction error, evaluated from the image obtained at the output layer. Once the dimension of the coding vector was determined, the encoder part containing the image feature information in the latent space was then used to extract the 1 × 49-dimension row vector written into the *.csv* file. After normalization processes, this was then appended with the input parameter data to obtain the final multimodal dataset, now containing data extracted from images and input parameter settings. The final prediction of weld quality was evaluated in this dataset. The obtained dataset was divided in a train-to-test ratio of 70:30, which gave the final training and test data dimensions as 19 × 53 and 9 × 53, where the LSS was chosen as the target variable for prediction. The prepared data were then subjected to a decision tree regression algorithm to predict the LSS values. The LSS value determines the weld quality of the USW joint.

The combined dataset was subjected to regression algorithms to predict LSS values. The results were evaluated using the mean absolute error value and RMSE values. The regression algorithm was first evaluated, and we found that the decision tree regressor gave lower prediction errors than the random forest regressor. The decision tree regressor was subjected to further parameter tuning to achieve minimum value for the function *neg-mean-squared-error* from the *GridSearchCV* function in Scikit Learn. Table 2 shows the values obtained using different parameters in the regression algorithm.

#### Discussion of Results

**Figure 11:**
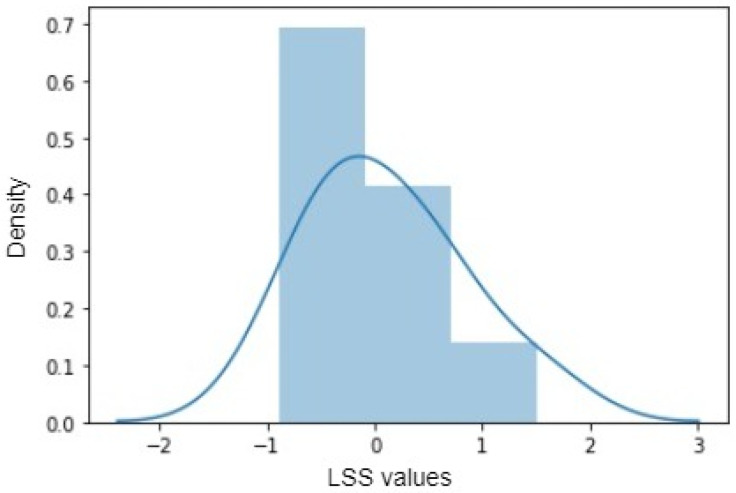
Difference between labelled target variable(LSS) and predicted LSS values.

The final prediction errors showed a 34% reduction in prediction errors when the image data were combined with the available input parameter data. This is backed by values in Table 3, which supports that data features extracted using CAE have helped considerably reduce the prediction errors in the case of an extremely limited dataset. Figure 11 shows the bell curve, which indicates that the predicted values are within the range of the original labeled data values.

### 4.4. Results from Weld Defect Detection

**Figure 12:**
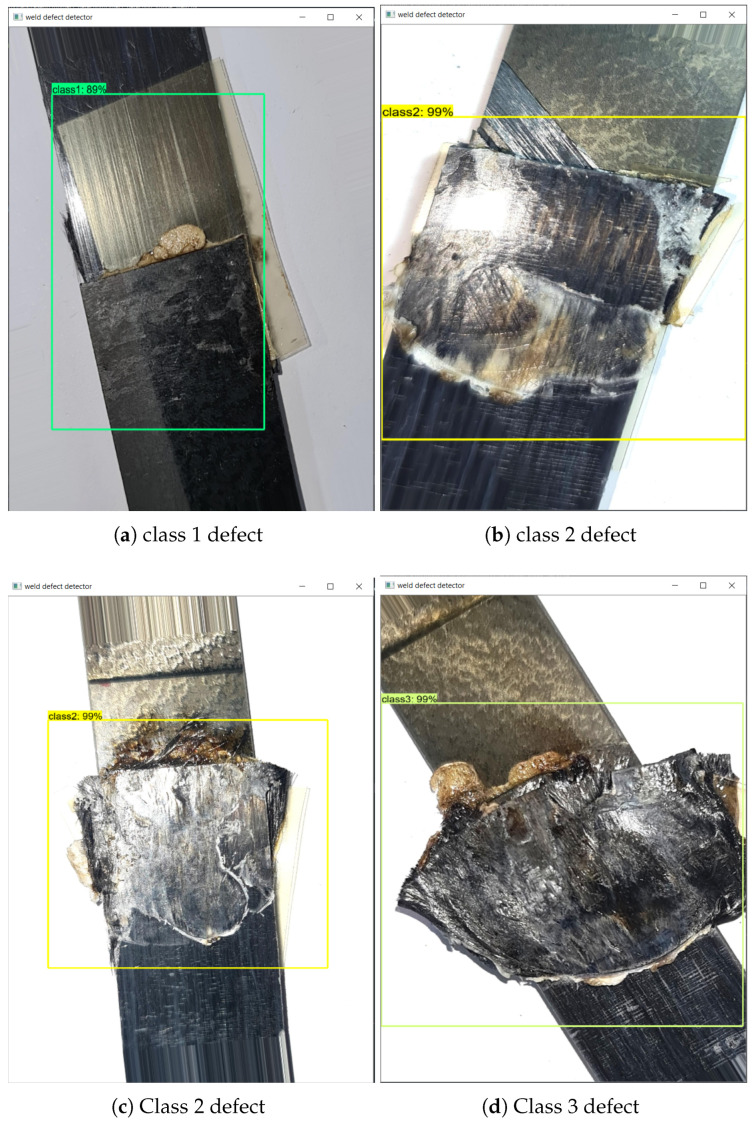
Detection results with high confidence (**a**) Class 1 (**b**,**c**) shows Class 2 (**d**) Class 3 defects.

**Figure 13:**
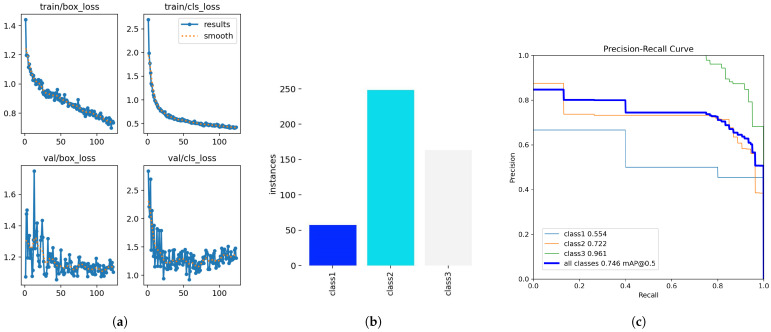
Training curves showing signs of overfitting, a plot showing number instances in each class, and precision-recall curve for USW defect dataset. (**a**) Training curves of yolov8n. (**b**) Plot showing no. of class instances. (**c**) Precision-Recall plot of the results.

The weld defect detection was conducted on the USW weld defect dataset set up in this experiment, and the detection results are shown in Figure 12. The deep learning models selected were trained on the augmented dataset, and further augmentation was applied during training. The training curves for YOLOv8n Figure 13a and the plot of the number of class instances Figure 13b in each class are given in Figure 13. The training loss decreased, but the validation losses increased, showing over-fitting. Though the training seemed to over-fit, the overall results are promising, indicating that image augmentation can be used to account for a low number of training image samples in a dataset.

The detection model, SSD-MobileNet (downloaded from the TensorFlow model zoo, part of TensorFlow Model Garden [82]), was trained using TensorFlow Object Detection API. The pretrained YOLOv8n and YOLOv8x models were used from YOLO-Ultralytics [76]. The detection showed promising results, with a high confidence score of >90% across all instances. The defect detection results have been compared with and without the augmentation of random noise and image-mosaics applied online during training. Results in Table 4 show that random noise augmentation added to the dataset further increased the defect detection accuracy. The YOLOv8x is the best and largest model among the YOLOv8 family, and YOLOv8n is the nano model, explicitly designed for mobile devices and edge processing devices. These results also validate that data augmentation helped improve the overall accuracy of the deep learning model.

Due to the limited sample size, the data available for validation were insufficient to expand the training and validation experiments further. Hence, the results were compared against weld defect detection using radiographic images available in the literature [1]. In [1], the authors applied augmentation on the original sample size of 95 images and divided images into patches to increase the dataset size for the segmentation task. The comparison of results is given in Table 5. The mAP we obtained in defect detection on our USW defect dataset was **0.74**, which was comparable to the mIoU **0.73** obtained for the segmentation using ResNet on the radiographic images of welding defects (RIWD) dataset [1]. However, this is not a direct comparison, as the tasks under consideration, object detection and segmentation, are significantly different to one another and also performed on different datasets. For better comparison, a publicly available surface defect detection dataset, NEU-DET, was used to repeat the training experiments with all the above-mentioned deep learning models. Table 4 compares metrics after training the publicly available weld defect dataset, NEU-DET, against the proposed USW defect dataset for object detection using YOLOv8. The classes in the two datasets are based on the defects found in the material used. Table 4 shows comparable performance for YOLOv8n against the YOLOv8x model; the YOLOv8x is known to be the most accurate yet slowest among multiple versions of the YOLOv8 models available. The YOLOv8x has 68.2 million parameters against the 3.2 million parameters in YOLOv8n.

#### Discussion of Results

Table 4 compares the accuracy metrics of YOLOv8n and YOLOv8x on the USW defect and NEU-DET datasets. The YOLOv8 models were trained for 200 epochs. However, YOLOv8 on the NEU-DET dataset was trained for 350 epochs because of the comparably larger size of the dataset. The models showed close accuracy values of **0.72** for YOLOv8x and **0.74** for YOLOv8n for the same augmentation parameters. The recall values were also close to one another: **0.82** for YOLOv8n and **0.83** for YOLOv8x, for the same augmentation parameters. However, the precision values on both datasets were lower in the same parameter setting: **0.54** for YOLOv8x and **0.55** for YOLOv8n.

**Figure 14:**
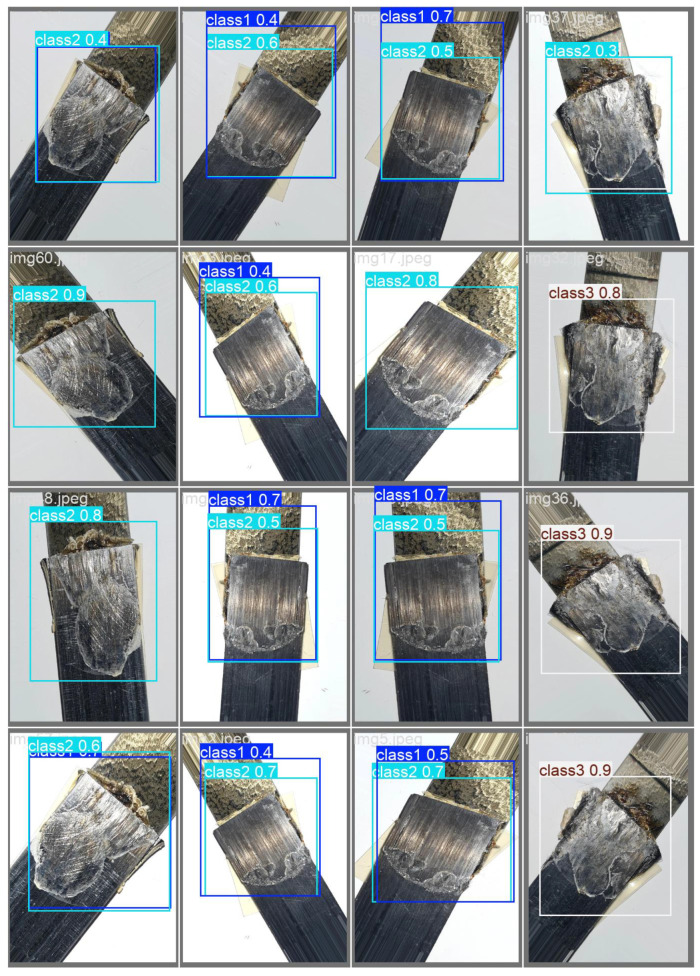
Misclassification in USW defect detection among class 1 and class 2.

**Figure 15:**
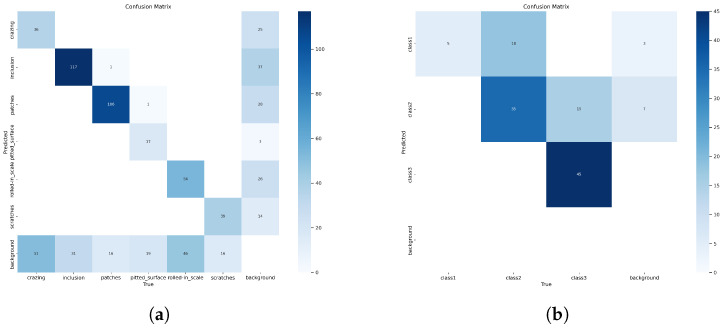
Confusion matrix showing detection results of YOLOv8x model on NEU-DET dataset and USW defect detection dataset. (**a**) Confusion matrix: NEU-DET. (**b**) Confusion matrix: USW defect dataset.

**Figure 16:**
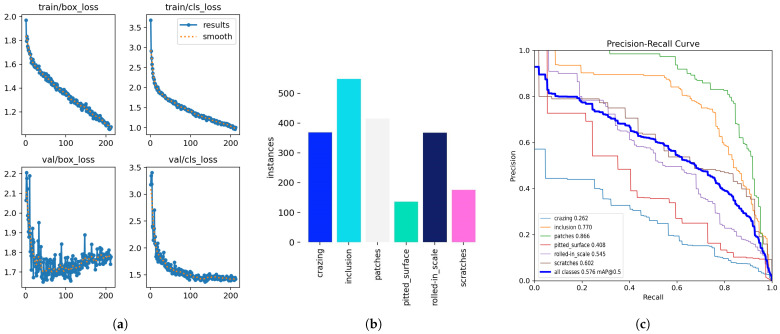
Training curves, plot showing number instances in each class, and precision-recall curve for NEU-DET dataset. (**a**) Training curves of NEU-DET. (**b**) Plot showing no. of class instances. (**c**) Precision-recall plot of the results.

The YOLOv8 detection models often misclassified classes 1 and 2 in the USW defect dataset, as seen in Figure 14. This is likely due to the imbalance in the number of class instances corresponding to these classes, as seen in Figure 13b. The data instances for class 1 were very low compared to the number of instances for classes 2 and 3. This imbalance in class 1 instances also resulted in lower classification scores. Consider the confusion matrix in Figure 15b; the number of instances of class 2 wrongly classified as class 1 was 18, but classes 2 and 3 showed promising results with more accurate classifications. Class 3 had the highest number of accurate classifications. The models, however, learned a clear distinction between classes 1 and 3, that no class 3 was misclassified as class 1. However, Figure 15a shows higher prediction accuracy for all 5 classes except the ’pitted_surface’ class. Figure 16b shows a comparably lower number of instances for the ’pitted_surface’ category. The training curves for the NEU-DET dataset, given in Figure 16a training, did not show signs of overfitting.

**Figure 17:**
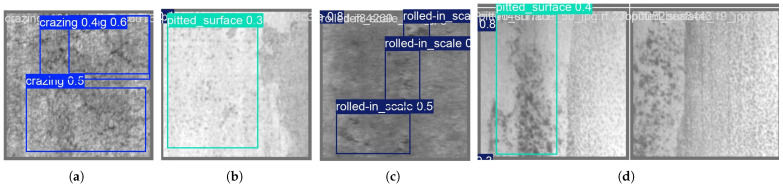
Detection results for NEU-DET surface defect dataset showing detection results for crazing, pitted surface and rolled-in-scale defects. (**a**) crazing. (**b**) pitted surface. (**c**) rolled-in-scale. (**d**) pitted surface-false negative case.

The NEU-DET dataset has a significantly higher number of training instances, 1800 in total, with each class represented by 300 images. The NEU-DET steel surface defect dataset consists of surface defects in steel. It is not essentially a weld defect detection dataset. Despite the higher number of representative images in the NEU-DET dataset, the mAP value after training was 0.59 on the YOLOv8n model. The precision value was 0.55, and the recall was 0.61 for the NEU-DET dataset on YOLOv8n. The training curves are given in Figure 16a, and the plot of the number of instances in each class is given in Figure 16b. The precision-recall curve for the results is given in Figure 16c in Figure 16. The precision-recall curves show the reduction in precision and recall values, as given in Table 4. Figure 17 shows the crazing defects detected in Figure 17a, pitted surface defects in Figure 17b, and the rolled-in-scale defects in Figure 17c. Figure 17d illustrates a case of missed detection for a pitted surface defect, which identifies the recall value as being low and highlights that the model does not detect all the instances in the ’pitted surface’ class that are present in this dataset. These results represent one particular case of false negatives, but the low recall value indicates further missed detections. However, the algorithm does ‘fail-safe’ in so much that a low recall value indicates that operator assistance is required to classify the defect in question accurately.

## 5. Conclusions

This work proposes a complete weld quality inspection process with a limited dataset. It proceeds with weld strength prediction using the generated multimodal dataset and visual defect detection using the developed USW weld defect detection dataset. This work is significant in determining the next downstream manufacturing process step based on a real-time assessment of USW joint quality.

The image data combined with labelled input parameter data were used to engineer a multimodal dataset that can be used to predict weld strength accurately. A significant reduction in detection error values (34%) coupled with a significant increase in prediction performance using the new augmented multimodal dataset was observed in comparison with the use of input parameter data only. A CAE training methodology that significantly lowers reconstruction errors for feature extraction has been proposed. The validity of the approach for extracting actionable downstream process information from images has been demonstrated. When combined with sensor information, the extracted data reduces error values during the prediction process, which is highly beneficial when extracting information in a setting where the cost of collection and augmentation of new data is prohibitive. The original data from the training specimens is then compressed, leading to efficient machine learning-based prediction using as few as 30 individual samples of good and bad joints.

The use of data augmentation to upgrade a constrained image dataset to generate valuable defect detection using computer vision algorithms is demonstrated in the second experiment. Offline model-specific augmentation techniques are presented that enable high-accuracy weld defect detection models to be developed. The detections achieved an overall mAP value of 0.74, which is a promising result, and image augmentation can be used to address the problem of limited datasets. Reliable classification using such a modest primary dataset has been shown to be appropriate for this use case. It leads to increased confidence that the probability of successful general deployment in a real-time setting will grow. This is particularly useful in engineering applications where the dataset is limited due to the cost associated with data generation.

In future work, general observations on the applicability of CAE feature extraction and its effect on subsequent predictions will be considered. Furthermore, autoencoder models like stacked autoencoders (for feature extraction) and the merits of variational autoencoders for data generation will be considered.

## Figures and Tables

**Table 1:** Comparison of the effect of longer training epochs on the overall prediction results.

No. of Epochs	Dimension of Bottleneck Layer	MAE	RMSE
60	7 × 7 × 1	6.83	8.67
80	7 × 7 × 1	6.89	7.82
100	7 × 7 × 1	6.93	8.70

**Table 2:** Parameter tuning for decision tree regression model to determine the best configuration for the lowest error values possible.

Decision Tree Regression-Parameter Tuning	MAE	MSE	RMSE
Max_depth = 10	7.7960	92.8485	9.6357
Max_depth = 10, Random_state = 0	8.6480	84.9982	9.2194
Max_depth = 10, Random_state = None	7.688	75.4092	8.6838
Max_depth = 5, Random_state = None	7.956	96.3131	9.8139
Max_depth = 3, Random_state = None	7.2493	61.2585	7.8267

**Table 3:** Comparison of the effect of multimodal training data on the overall prediction results.

	Data Used for Final Prediction	MAE (Final)	RSME (Final)
1	Image features extracted (CAE)	10.98	11.98
2	Image features data + input param, data	**7.24**	**7.82**

**Table 4:** Performance comparison of YOLOv8n vs. YOLOv8x on USW defect detection dataset vs. NEU-DET surface defect dataset.

Dataset	Model	Precision	Recall	mAP50	mAP50-95
USW-defect dataset	YOLOv8n	0.61	0.84	0.61	0.43
USW-defect dataset (Aug:noise:0.1)	YOLOv8n	0.66	0.80	0.68	0.43
USW-defect dataset (Aug:noise:0.3)	YOLOv8n	0.55	**0.82**	**0.746**	**0.50**
USW-defect dataset	YOLOv8x	0.65	0.82	0.79	0.58
USW-defect dataset (Aug:noise:0.3)	YOLOv8x	0.64	**0.83**	**0.72**	**0.52**
NEU-DET	YOLOv8x	0.54	0.61	0.58	0.28
NEU-DET	YOLOv8n	**0.55**	0.61	**0.59**	0.29
NEU-DET (Aug:noise:0.3)	YOLOv8n	0.52	0.60	0.57	0.29

Note: Numbers in Red indicate high Recall, mAP50 and mAP50-95 values for USW dataset. Numbers in Blue indicate values from NEU-DET dataset.

**Table 5:** Comparison of defect detection result in literature with segmentation result on a limited dataset with <100 original sample size.

Data Used for Final Prediction	Original Sample Size	Accuracy	Recall
USW defect dataset	28	**0.74** (mAP)	0.82
Radiographic images of welding defects (RIWD)	95	0.73 (mIoU)	**0.86**

## Data Availability

The authors confirm that the data supporting the findings of this study are available within the article.

## Abbreviations

The following abbreviations are used in this manuscript:

CNN	Convolutional Neural Network
CAE	Convolutional Autoencoder
IoT	Internet of Things
LSS	Lap Shear Strength
MAE	Mean Absolute Error MAE
NDT	Non-Destructive Testing
NDI	Non-Destructive Inspection
NEU	North Eastern University
PCA	Principal Component Analysis
RGB	red-green-blue
RMSE	Root Mean-Squared Error
SSIM	Structural Similarity Index Measure
USW	Ultrasonic Welding

## References

[1] Xu H., Yan Z., Ji B., Huang P., Cheng J., Wu X. (2022). Defect detection in welding radiographic images based on semantic segmentation methods. Measurement.

[2] Hou W., Zhang D., Wei Y., Guo J., Zhang X. (2020). Review on computer-aided weld defect detection from radiography images. Appl. Sci..

[3] Liao T.W. (2009). Improving the accuracy of computer-aided radiographic weld inspection by feature selection. NDT E Int..

[4] Zuo F., Liu J., Zhao X., Chen L., Wang L. (2023). An X-ray-based automatic welding defect detection method for special equipment system. IEEE/ASME Trans. Mechatron..

[5] Stavridis J., Papacharalampopoulos A., Stavropoulos P. (2018). Quality assessment in laser welding: A critical review. Int. J. Adv. Manuf. Technol..

[6] Reddy K.A. (2017). Non-destructive testing, evaluation of stainless steel materials. Mater. Today Proc..

[7] Block S.B., Da Silva R.D., Lazzaretti A.E., Minetto R. (2024). LoHi-WELD: A novel industrial dataset for weld defect detection and classification, a deep learning study, and future perspectives. IEEE Access.

[8] Nadzri N.A., Ishak M., Saari M.M., Halil A.M. Development of eddy current testing system for welding inspection. Proceedings of the 2018 9th IEEE Control and System Graduate Research Colloquium (ICSGRC).

[9] LeTessier R., Coade R., Geneve B. (2002). Sizing of cracks using the alternating current field measurement technique. Int. J. Press. Vessel. Pip..

[10] Madhvacharyula A.S., Pavan A.V.S., Gorthi S., Chitral S., Venkaiah N., Kiran D.V. (2022). In situ detection of welding defects: A review. Weld. World.

[11] Handoko D. (2023). Weld Defect Detection and Classification based on Deep Learning Method: A Review. J. Ilmu Komput. Dan Inf. J. Comput. Sci. Inf..

[12] Li P., Pei Y., Li J. (2023). A comprehensive survey on design and application of autoencoder in deep learning. Appl. Soft Comput..

[13] Xu M., Yoon S., Fuentes A., Park D.S. (2023). A comprehensive survey of image augmentation techniques for deep learning. Pattern Recognit..

[14] Mongan P.G., Hinchy E.P., O’Dowd N.P., McCarthy C.T. (2021). Quality prediction of ultrasonically welded joints using a hybrid machine learning model. J. Manuf. Process..

[15] Mongan P.G., Modi V., McLaughlin J.W., Hinchy E.P., O’Higgins R.M., O’Dowd N.P., McCarthy C.T. (2022). Multi-objective optimisation of ultrasonically welded dissimilar joints through machine learning. J. Intell. Manuf..

[16] Rizvi S.A., Alib W. (2019). Welding defects, Causes and their Remedies: A Review: Welding defects. Teknomekanik.

[17] Benatar A., Marcus M. (2023). Ultrasonic welding of plastics and polymeric composites. Power Ultrasonics.

[18] Rajakumar S., Kavitha S., Sonar T. (2023). Optimization of ultrasonic welding parameters to maximize the tensile shear fracture load bearing capability of lap welded ABS plastic sheets. Int. J. Interact. Des. Manuf. IJIDeM.

[19] Jones I. (2013). Laser welding of plastics. Handbook of Laser Welding Technologies.

[20] Bindal T., Saxena R.K., Pandey S. (2021). Investigating friction spin welding of thermoplastics in shear joint configuration. SN Appl. Sci..

[21] Stokes V., Hobbs S. (1993). Vibration welding of ABS to itself and to polycarbonate, poly (butylene terephthalate), poly (ether imide) and modified poly (phenylene oxide). Polymer.

[22] Bucknall C., Drinkwater I., Smith G. (1980). Hot plate welding of plastics: Factors affecting weld strength. Polym. Eng. Sci..

[23] Trofimov N., Yulenets Y.P., Markov A. (2011). High-frequency welding of plastic components of complicated shape. Weld. Int..

[24] Tiwary V.K., Padmakumar A., Malik V. (2022). Adhesive bonding of similar/dissimilar three-dimensional printed parts (ABS/PLA) considering joint design, surface treatments, and adhesive types. Proc. Inst. Mech. Eng. Part C J. Mech. Eng. Sci..

[25] Tripicchio P., Camacho-Gonzalez G., D’Avella S. (2020). Welding defect detection: Coping with artifacts in the production line. Int. J. Adv. Manuf. Technol..

[26] Hosseinabad E.R., Zaman M.A.U. (2020). A brief review on cellular manufacturing and group technology. Res. J. Manag. Rev..

[27] Chen Y., Ding Y., Zhao F., Zhang E., Wu Z., Shao L. (2021). Surface defect detection methods for industrial products: A review. Appl. Sci..

[28] Ördek B., Borgianni Y., Coatanea E. (2024). Machine learning-supported manufacturing: A review and directions for future research. Prod. Manuf. Res..

[29] Wang J., Ma Y., Zhang L., Gao R.X., Wu D. (2018). Deep learning for smart manufacturing: Methods and applications. J. Manuf. Syst..

[30] Ahmad H.M., Rahimi A. (2022). Deep learning methods for object detection in smart manufacturing: A survey. J. Manuf. Syst..

[31] Cumbajin E., Rodrigues N., Costa P., Miragaia R., Frazão L., Costa N., Fernández-Caballero A., Carneiro J., Buruberri L.H., Pereira A. (2023). A systematic review on deep learning with CNNs applied to surface defect detection. J. Imaging.

[32] Givnan S., Chalmers C., Fergus P., Ortega-Martorell S., Whalley T. (2022). Anomaly detection using autoencoder reconstruction upon industrial motors. Sensors.

[33] Liang H., Fu W., Yi F. A survey of recent advances in transfer learning. Proceedings of the 2019 IEEE 19th International Conference on Communication Technology (ICCT).

[34] Guo W., Qu H., Liang L. WDXI: The dataset of X-ray image for weld defects. Proceedings of the 2018 14th International Conference on Natural Computation, Fuzzy Systems and Knowledge Discovery (ICNC-FSKD).

[35] Papageorgiou E.I., Theodosiou T., Margetis G., Dimitriou N., Charalampous P., Tzovaras D., Samakovlis I. Short survey of artificial intelligent technologies for defect detection in manufacturing. Proceedings of the 2021 12th International Conference on Information, Intelligence, Systems & Applications (IISA).

[36] Jeffers J., Reinders J. (2013). Intel Xeon Phi Coprocessor High Performance Programming.

[37] Sassi P., Tripicchio P., Avizzano C.A. (2019). A smart monitoring system for automatic welding defect detection. IEEE Trans. Ind. Electron..

[38] Huang G., Liu Z., Van Der Maaten L., Weinberger K.Q. Densely connected convolutional networks. Proceedings of the IEEE Conference on Computer Vision and Pattern Recognition.

[39] Le X., Mei J., Zhang H., Zhou B., Xi J. (2020). A learning-based approach for surface defect detection using small image datasets. Neurocomputing.

[40] Martin A., Soumith C., Leon B. Wasserstein generative adversarial networks. Proceedings of the International Conference on Machine Learning.

[41] Szegedy C., Vanhoucke V., Ioffe S., Shlens J., Wojna Z. Rethinking the inception architecture for computer vision. Proceedings of the IEEE Conference on Computer Vision and Pattern Recognition.

[42] Howard A.G., Zhu M., Chen B., Kalenichenko D., Wang W., Weyand T., Andreetto M., Adam H. (2017). MobileNets: Efficient Convolutional Neural Networks for Mobile Vision Applications. arXiv.

[43] Gao Y., Lin J., Xie J., Ning Z. (2020). A real-time defect detection method for digital signal processing of industrial inspection applications. IEEE Trans. Ind. Inform..

[44] He Y., Song K., Meng Q., Yan Y. (2019). An end-to-end steel surface defect detection approach via fusing multiple hierarchical features. IEEE Trans. Instrum. Meas..

[45] Tang S., He F., Huang X., Yang J. (2019). Online PCB defect detector on a new PCB defect dataset. arXiv.

[46] Yang L., Fan J., Huo B., Liu Y. (2021). Inspection of welding defect based on multi-feature fusion and a convolutional network. J. Nondestruct. Eval..

[47] Tao X., Wang Z., Zhang Z., Zhang D., Xu D., Gong X., Zhang L. (2018). Wire defect recognition of spring-wire socket using multitask convolutional neural networks. IEEE Trans. Compon. Packag. Manuf. Technol..

[48] Alfaro S.C., Franco F.D. (2010). Exploring infrared sensoring for real time welding defects monitoring in GTAW. Sensors.

[49] Duan F., Yin S., Song P., Zhang W., Zhu C., Yokoi H. (2019). Automatic welding defect detection of x-ray images by using cascade adaboost with penalty term. IEEE Access.

[50] Roy R.B., Ghosh A., Bhattacharyya S., Mahto R.P., Kumari K., Pal S.K., Pal S. (2018). Weld defect identification in friction stir welding through optimized wavelet transformation of signals and validation through X-ray micro-CT scan. Int. J. Adv. Manuf. Technol..

[51] Li Y., Li Y.F., Wang Q.L., Xu D., Tan M. (2009). Measurement and defect detection of the weld bead based on online vision inspection. IEEE Trans. Instrum. Meas..

[52] Chen B., Fang Z., Xia Y., Zhang L., Huang Y., Wang L. (2018). Accurate defect detection via sparsity reconstruction for weld radiographs. NDT E Int..

[53] Du D., Cai G.R., Tian Y., Hou R.S., Wang L. (2007). Automatic inspection of weld defects with x-ray real-time imaging. Robotic Welding, Intelligence and Automation.

[54] Günther J., Pilarski P.M., Helfrich G., Shen H., Diepold K. (2016). Intelligent laser welding through representation, prediction, and control learning: An architecture with deep neural networks and reinforcement learning. Mechatronics.

[55] Yang L., Liu Y., Peng J. (2019). An automatic detection and identification method of welded joints based on deep neural network. IEEE Access.

[56] Yang J., Fu G., Zhu W., Cao Y., Cao Y., Yang M.Y. (2020). A deep learning-based surface defect inspection system using multiscale and channel-compressed features. IEEE Trans. Instrum. Meas..

[57] Iandola F.N., Han S., Moskewicz M.W., Ashraf K., Dally W.J., Keutzer K. (2016). SqueezeNet: AlexNet-level accuracy with 50x fewer parameters and < 0.5 MB model size. arXiv.

[58] Yi L., Li G., Jiang M. (2017). An end-to-end steel strip surface defects recognition system based on convolutional neural networks. Steel Res. Int..

[59] Fu G., Sun P., Zhu W., Yang J., Cao Y., Yang M.Y., Cao Y. (2019). A deep-learning-based approach for fast and robust steel surface defects classification. Opt. Lasers Eng..

[60] Shorten C., Khoshgoftaar T.M. (2019). A survey on image data augmentation for deep learning. J. Big Data.

[61] Yang J., Bai Y., Li G., Liu M., Liu X. (2015). A novel method of diagnosing premature ventricular contraction based on sparse auto-encoder and softmax regression. Bio-Med Mater. Eng..

[62] Goodfellow I., Bengio Y., Courville A. (2016). Deep Learning.

[63] Maggipinto M., Masiero C., Beghi A., Susto G.A. (2018). A convolutional autoencoder approach for feature extraction in virtual metrology. Procedia Manuf..

[64] Gong D., Liu L., Le V., Saha B., Mansour M.R., Venkatesh S., Hengel A.v.d. Memorizing normality to detect anomaly: Memory-augmented deep autoencoder for unsupervised anomaly detection. Proceedings of the IEEE/CVF International Conference on Computer Vision.

[65] Intro to Autoencoders. https://www.tensorflow.org/tutorials/generative/autoencoder.

[66] Toğaçar M., Ergen B., Cömert Z. (2020). Application of breast cancer diagnosis based on a combination of convolutional neural networks, ridge regression and linear discriminant analysis using invasive breast cancer images processed with autoencoders. Med. Hypotheses.

[67] Cui X., Liu S., Lin Z., Ma J., Wen F., Ding Y., Yang L., Guo W., Feng X. (2021). Two-step electricity theft detection strategy considering economic return based on convolutional autoencoder and improved regression algorithm. IEEE Trans. Power Syst..

[68] Steck H. (2020). Autoencoders that don’t overfit towards the identity. Adv. Neural Inf. Process. Syst..

[69] Lončarević Z., Gams A., Ude A. (2021). Robot skill learning in latent space of a deep autoencoder neural network. Robot. Auton. Syst..

[70] Dillon B.M., Plehn T., Sauer C., Sorrenson P. (2021). Better latent spaces for better autoencoders. SciPost Phys..

[71] Yu S., Principe J.C. (2019). Understanding autoencoders with information theoretic concepts. Neural Netw..

[72] Tapia N.I., Estévez P.A. On the information plane of autoencoders. Proceedings of the 2020 International Joint Conference on Neural Networks (IJCNN).

[73] Hore A., Ziou D. Image quality metrics: PSNR vs. SSIM. Proceedings of the 2010 20th International Conference on Pattern Recognition.

[74] Ndajah P., Kikuchi H., Yukawa M., Watanabe H., Muramatsu S. SSIM image quality metric for denoised images. Proceedings of the 3rd WSEAS International Conference on Visualization, Imaging and Simulation.

[75] Pawłowski M., Wróblewska A., Sysko-Romańczuk S. (2023). Effective techniques for multimodal data fusion: A comparative analysis. Sensors.

[76] Jocher G., Chaurasia A., Qiu J. (2023). Ultralytics YOLOv8. https://docs.ultralytics.com/.

[77] Hendrycks D., Lee K., Mazeika M. Using pre-training can improve model robustness and uncertainty. Proceedings of the International Conference on Machine Learning, PMLR.

[78] Li H., Singh B., Najibi M., Wu Z., Davis L.S. (2019). An analysis of pre-training on object detection. arXiv.

[79] Sun W., Zhao H., Jin Z. (2017). An efficient unconstrained facial expression recognition algorithm based on stack binarized auto-encoders and binarized neural networks. Neurocomputing.

[80] Akbiyik M.E. (2023). Data Augmentation in Training CNNs: Injecting Noise to Images. arXiv.

[81] Hao W., Zhili S. (2020). Improved Mosaic: Algorithms for more Complex Images. J. Physics Conf. Ser..

[82] Yu H., Chen C., Du X., Li Y., Rashwan A., Hou L., Jin P., Yang F., Liu F., Kim J. (2020). TensorFlow Model Garden. https://github.com/tensorflow/models.

